# Expression profiling of lncRNAs and mRNAs in placental site trophoblastic tumor (PSTT) by microarray

**DOI:** 10.7150/ijms.65002

**Published:** 2022-01-01

**Authors:** Jianfeng Gan, Zhixian Chen, Xuan Feng, Zhi Wei, Sai Zhang, Yan Du, Congjian Xu, Hongbo Zhao

**Affiliations:** 1 Shanghai Key Laboratory of Female Reproductive Endocrine Related Diseases, Obstetrics and Gynecology Hospital, Fudan University, Shanghai 200011, People's Republic of China.; 2 Department of Obstetrics and Gynecology of Shanghai Medical School, Fudan University, Shanghai 200032, People's Republic of China.

**Keywords:** placental site trophoblastic tumor (PSTT), microarray analysis, lncRNA

## Abstract

As a rare type of gestational trophoblastic disease, placental site trophoblastic tumor (PSTT) is originated from intermediate trophoblast cells. Long noncoding RNAs (lncRNAs) regulate numerous biological process. However, the role of lncRNAs in PSTT remains poorly understood. In the present study, expression levels of lncRNAs and mRNAs in four human PSTT tissues and four normal placental villi were investigated. The results of microarray were validated by the reverse transcription and quantitative real-time polymerase reaction (RT-qPCR) and immunohistochemistry analyses. Furthermore, GO and KEGG pathway analyses were performed to identify the underlying biological processes and signaling pathways of aberrantly expressed lncRNAs and mRNAs. We also conducted the coding-non-coding gene co-expression (CNC) network to explore the interaction of altered lncRNAs and mRNAs. In total, we identified 1247 up-regulated lncRNAs and 1013 down-regulated lncRNAs as well as 828 up-regulated mRNAs and 1393 down-regulated mRNAs in PSTT tissues compared to normal villi (fold change ≥ 2.0, p < 0.05). GO analysis showed that mitochondrion was the most significantly down-regulated GO term, and immune response was the most significantly up-regulated term. A CNC network profile based on six confirmed lncRNAs (NONHSAT114519, NR_103711, NONHSAT003875, NONHSAT136587, NONHSAT134431, NONHSAT102500) as well as 354 mRNAs was composed of 497 edges. GO and KEGG analyses indicated that interacted mRNAs were enriched in the signal-recognition particle (SRP)-dependent cotranslational protein targeting to membrane and Ribosome pathway. It contributes to expand the understanding of the aberrant lncRNAs and mRNAs profiles of PSTT, which may be helpful for the exploration of new diagnosis and treatment of PSTT.

## Introduction

Placental site trophoblastic tumor (PSTT) is a rare type of gestational trophoblastic neoplasms (GTN), which was first described in 1976^1^. Compared with other types of GTN, PSTT tends to have a more unpredictable biological behavior, chemotherapy resistance and poor prognosis 2. Therefore, hysterectomy is the first-consideration for PSTT patients who may face the fertility problems after the operations 3. The proportion of PSTT in GTN is about 0.2-3% with an estimated incidence of 1/100000 pregnancies 4-10. The early diagnosis of PSTT remains unclear, and distinguishing the benign and malignant forms of PSTT in the early stage is important to determine next clinical therapy 11. Therefore, it is in urgent need for a better comprehension of PSTT to explore new diagnostic and therapeutic targets.

Long non-coding RNAs (lncRNAs) are non-protein-coding RNAs that longer than 200 nucleotides, which have been confirmed to function as regulators of many cellular processes, such as development, differentiation, cell fate and disease pathogenesis as well as tumorigenesis 12-14. It has been reported that lncRNAs may play important roles in chemoresistance in many malignant tumors including ovarian cancer 15, breast cancer 16, gastric cancer 17, glioblastoma 18. For example, LINC00261 is abnormally expressed in a number of tumors including pancreatic cancer, gastric cancer, choriocarcinoma and so on 19. It has been confirmed to alleviate cisplatin resistance in colon-cancer via Wnt/β-catenin pathway 20. Nevertheless, little is known about the lncRNA and mRNA expression profiles of PSTT and their involvement in the progression of PSTT. The present study performed the first microarray analysis of lncRNA and mRNA expression profiles of PSTT, which may contribute to expand our understanding of PSTT as well as explore more effective treatment strategy.

## Materials and methods

### Tissues and ethics statement

In total, 4 human first-trimester placental villi, 18 PSTT tissues were obtained from the tissue bank of Obstetrics and Gynecology Hospital of Fudan University. Written informed consent was obtained. The pathological diagnosis of choriocarcinoma and PSTT was confirmed in the department of pathology in Obstetrics and Gynecology Hospital. Human first-trimester placental villi (6-7 weeks of gestation) were collected from 25-30 years old women without underlying health conditions that had normal pregnancies and terminated for non-medical reasons. PSTT tissues were collected from surgeries for primary tumors. Patients were 25.5-38 years old with at least 1 gravidity and 1 parity. And the stage of PSTT is FIGO Ⅰ. All the PSTT tissues were affirmed by frozen sections to contain more than 75% lesions. This study was approved by the Institution Ethics Committee of Obstetrics and Gynecology Hospital of Fudan University.

### Microarray analysis

Total RNAs were isolated by using Trizol RNA extraction kit (Invitrogen, Carlsbad,CA, USA) and was quantified by the NanoDrop ND-2000 (Thermo Scientific), and the RNAs integrity and 28S/18S were assessed using Agilent Bioanalyzer 2100 (Agilent Technologies). The sample labeling, microarray hybridization and washing were performed based on the manufacturer's standard protocols. Briefly, total RNAs were transcribed to double strand cDNAs and then synthesized cRNAs. Next, 2nd cycle cDNAs were synthesized from cRNAs. Followed fragmentation and biotin labeling, the 2nd cycle cDNAs were hybridized onto the microarray. After washing and staining, the arrays were scanned by the Affymetrix Scanner 3000 (Affymetrix). The sample with RIN (RNA integrity number) values of RIN ≥ 7.0, 28S/18S ≥ 0.7 and the total amount can meet 2 or more experiments were processed for subsequent experiments. In this case, 4 human first-trimester placental villi and 4 PSTT tissues were selected and subjected to the Affymetrix Human OElncRNA Array.

### Reverse transcription and quantitative real-time polymerase chain reaction (RT-qPCR)

Quantification was performed with a two-step reaction process: reverse transcription (RT) and quantitative PCR. Firstly, mRNA was transcribed to cDNA in a GeneAmp® PCR System 9700 (Applied Biosystems, USA). The second step was performed in a GeneAmp® PCR System 9700 (Applied Biosystems, USA) with 5 × HiScript II Q RT SuperMix IIa. Quantitative Real-time PCR was performed using LightCycler® 480 Ⅱ Real-time PCR Instrument (Roche, Swiss). The primer sequences were designed and synthesized by Generay Biotech (Generay, PRC) based on the mRNA sequences obtained from the NCBI database as shown in Table 1.

### Immunohistochemistry

The first-trimester placental villi, choriocarcinoma tissues, and PSTT tissues sections were immunohistochemically stained by using primary antibodies of PLAC8, EGR1 and ADAMTS6 at 4°C overnight followed by the secondary antibody for 1 h at 37°C. The band was then visualized using the EnVision Detection Systems (Dako, Glostrup, Denmark). Paraffin-embedded tissue sections were stained with hematoxylin-eosin (HE) on Leica automated Staining/Coverslipping Workstations (Leica Biosystems, Nussloch, Germany). PLAC8 antibody (12284- 1-AP) for immunohistochemistry (IHC) was purchased from Proteintech (Chicago, IL, USA). EGR1 antibody (4154S) was purchased from Cell Signaling Technology (San Diego, CA, USA). ADAMTS6 antibody (PA5-60365) was obtained from Thermo Fisher Scientific (Waltham, MA, USA). HRP- conjugated secondary antibodies were purchased from Jackson ImmunoResearch Laboratories (West Grove, PA, USA).

### Gene Oncology (GO) analysis, Kyoto Encyclopedia of Genes and Genomes (KEGG) pathway analysis and coding-non-coding gene co-expression (CNC) network

GO analysis and KEGG analysis were applied to determine the potential biological functions and significant pathways of altered mRNAs or lncRNA-interacted associated mRNAs. Afterwards, we established a CNC network between validated 6 lncRNAs and interacted mRNAs to explore their relationships. Pearson correlation coefficients (PCCs) ≥ 0.95 were selected as the baseline of correlation analysis. Cytoscape V3.8.0 (The Cytoscape Consortium, San Diego, CA, USA) was used to represent the interaction in pictures.

### Gene set enrichment analysis (GSEA)

GSEA was conducted using the Molecular Signatures Database hallmark gene set collection (v7.4) to identify differences in pathways among choriocarcinoma (CC), PSTT, epithelioid trophoblastic tumor (ETT) and normal villi. Data set GSE135727 21 was downloaded from Gene Expression Omnibus 22.

### Statistical analysis

Affymetrix GeneChip Command Console (version 4.0, Affymetrix) software was used to extract raw data. Next, Expression Console (version1.3.1, Affymetrix) software offered RMA normalization for both gene and exon level analysis. Genesrping software (version 13.1; Agilent Technologies) was employed to finish the basic analysis. Differentially expressed genes were then identified through fold change as well as P value calculated with t-test. P values were also adjusted for multiple comparison. The threshold set for up- and down-regulated genes was fold change ≥ 2.0 and P value ≤ 0.05. Hierarchical Clustering was performed to display the distinguishable genes' expression pattern among samples.

## Results

### Expression profiles of lncRNAs and mRNAs in PSTT

**Figure 1:**
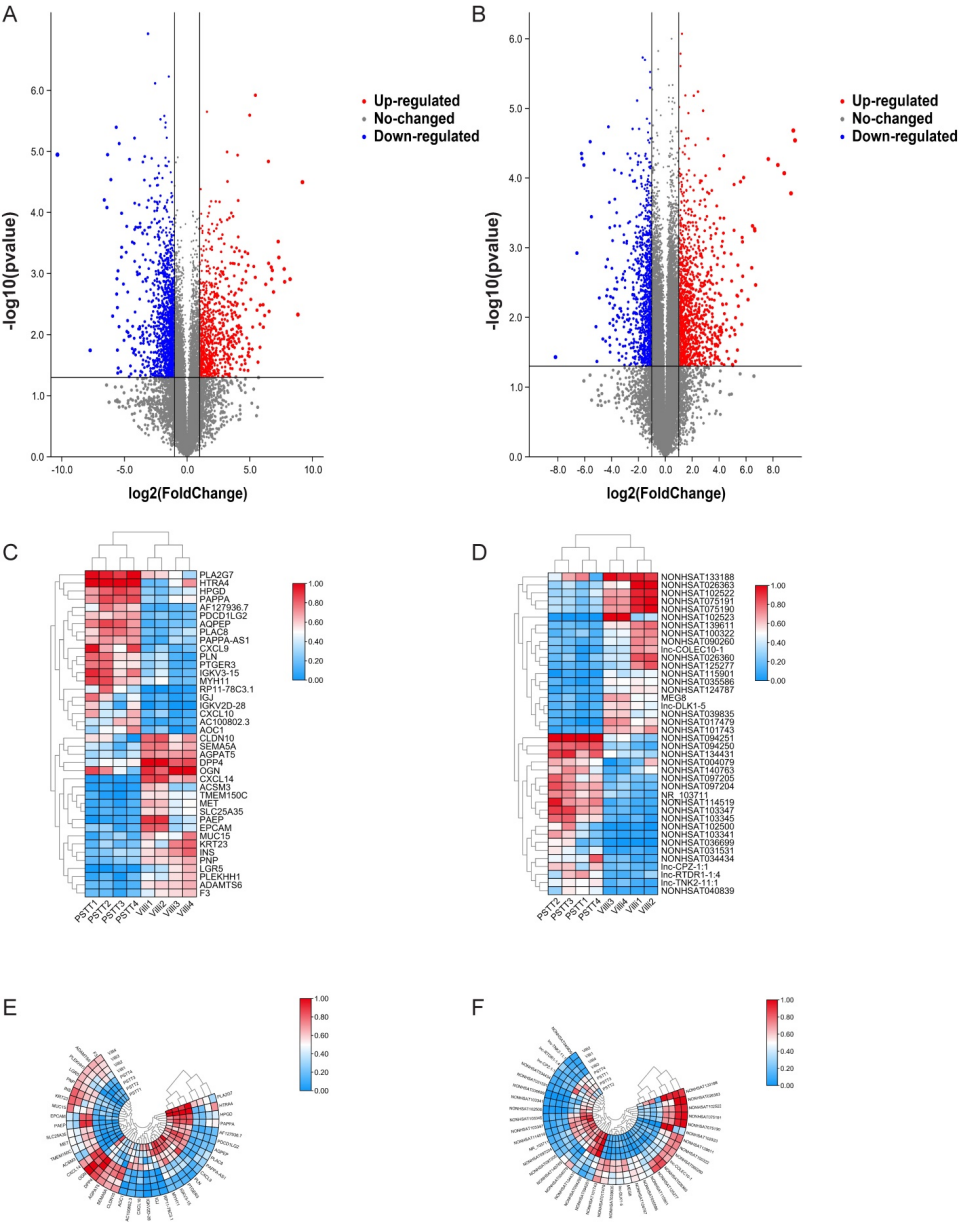
** Differential mRNA and lncRNA expression of PSTT and villi by microarray.** The volcano plot represents total identified mRNAs (A) and lncRNAs (B) expression between the PSTT and normal villi. The horizontal black lines show the default 2.0-fold change and the vertical black lines represent a P-value of 0.05. The red and blue plots represent up- and downregulated RNAs with FC ≥ 2.0 and P < 0.05. Heat map and hierarchical clustering analysis of the top 20 up- and downregulated mRNAs (C, E) and lncRNAs (D, F) between PSTT and normal villi.

In order to identify the expression of lncRNAs in PSTT compared to normal villi, microarray was conducted as described. Principal component analysis (PCA) was performed to explore the expression patterns associated with differentially expressed lncRNAs and mRNAs in our datasets. Both mRNA and lncRNA expression levels could be distinguished between the PSTT and control samples (Figure S1A and S1B). In brief, 1247 up-regulated lncRNAs and 1013 down-regulated lncRNAs were detected (FC ≥ 2.0 and P ≤ 0.05). The top 20 up-regulated and down-regulated lncRNAs are shown in Table 2. Among our data, the most significantly up-regulated lncRNA is NONHSAT114519 with FC of 801.27435, whereas NONHSAT102523 is the most significantly down-regulated lncRNA. Clustering analysis of top 20 up- and down-regulated lncRNAs and volcano plot of lncRNAs expression were shown in Figure 1D, F and B.

We also demonstrated 828 up-regulated mRNAs and 1393 down-regulated mRNAs in PSTT tissues compared to normal villi (fold change ≥ 2.0, p ≤ 0.05). The top 20 up-regulated and down-regulated mRNAs are shown in Table 3. Among those upregulated mRNAs, AQPEP also known as LVRN ranges as top with an FC of 591.0085, while expression of CXCL14 tops among downregulated genes with FC of 1304.5027. The heatmap plot showed the clustering analysis among mRNA expression profiles by revealing the top 20 up- and downregulated mRNAs (Figure 1 C, E). A volcano plot (Figure 1A) showed the variation of mRNA expression between PSTT tissues and normal villi. The heatmaps and hierarchical clustering were drawn by the TBtools 23.

### Validation of the aberrantly expressed lncRNAs and mRNAs by RT-qPCR

**Figure 2:**
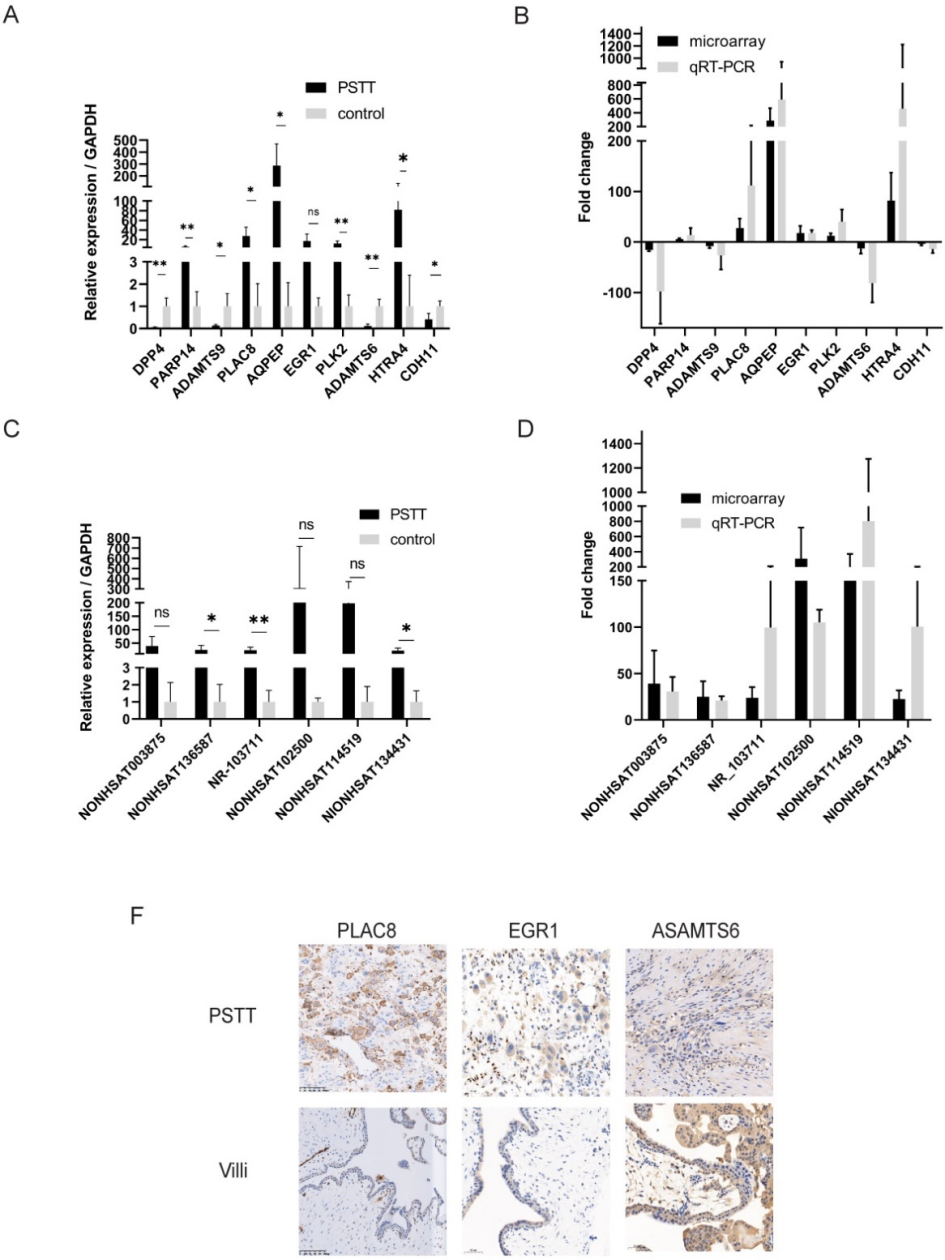
** Validation of the microarray results of mRNAs and lncRNAs by RT-qPCR and immunohistochemistry**. RT-qPCR was performed to test the differentially expressed mRNAs (A) and lncRNAs (C) (n=4 for PSTT group and control group, respectively); the fold change of each mRNA (B) and lncRNA (D) between PSTT tissues and control group was determined microarray and RT-qPCR. The expression of PLAC8 and EGR1 was obviously higher than normal villi, while ADAMTS6 expression is much lower in PSTT compared to normal villi (F). ns, No significance; *, P < 0.05; **, P < 0.01, P < 0.001, *Student t*-test. Scale bar = 100μm.

To confirm the reliability of the microarray profiling results, six lncRNAs (NONHSAT114519, NR_103711, NONHSAT003875, NONHSAT136587, NONHSAT134431, NONHSAT102500) as well as ten mRNAs *(DPP4*,* PARP14*,* ADAMTS9*,* PLAC8*, *AQPEP*,* EGR1*,* PLK2*,* ADAMTS6*,* HTRA4*,* CDH11*) were chosen randomly for RT-qPCR. The microarray samples including four PSTT tissues and normal villi counterparts were further used for RT-qPCR. The results of RT-qPCR were in accordance with microarray observations. In PSTT tissues, the lncRNA expression of NONHSAT114519, NR_103711, NONHSAT003875, NONHSAT136587, NONHSAT134431, NONHSAT102500 was up-regulated when compared with those in normal placental villi (Figure 2C, D). Further, the mRNA expression of *PARP14*, *PLAC8*, *AQPEP*, *EGR1*, *PLK2*, and* HTRA4* was significantly up-regulated whereas the expression of *DPP4*, *ADAMTS9*, *ADAMTS6* and *CDH11* in PSTT tissues was significantly down-regulated compared with those in normal placental villi (Figure 2A, B).

### Validation of the PLAC8, EGR1 and ADAMTS6 by immunohistochemistry

To further confirm the microarray data, the protein products of several genes were measured by immunohistochemistry analysis. We randomly chose two up-regulated mRNAs (*PLAC8* and *EGR1*) as well as one down-regulated mRNAs (*ADAMTS6*) for immunohistochemistry. As shown in Figure 2F, PLAC8 and EGR1 expression was dramatically elevated in PSTT tissues compared with those in normal villi. Comparatively, ADAMTS6 was diffusely positive in normal villi but only partly positive in PSTT. Immunohistochemistry data were consistent with microarray analysis. Furthermore, we also found that PLAC8 was highly expressed in PSTT but not choriocarcinoma, which indicates that PLAC8 may act as a potential marker to distinguish PSTT from choriocarcinoma Figure S2.

### GO and KEGG analyses of differential genes

**Figure 3:**
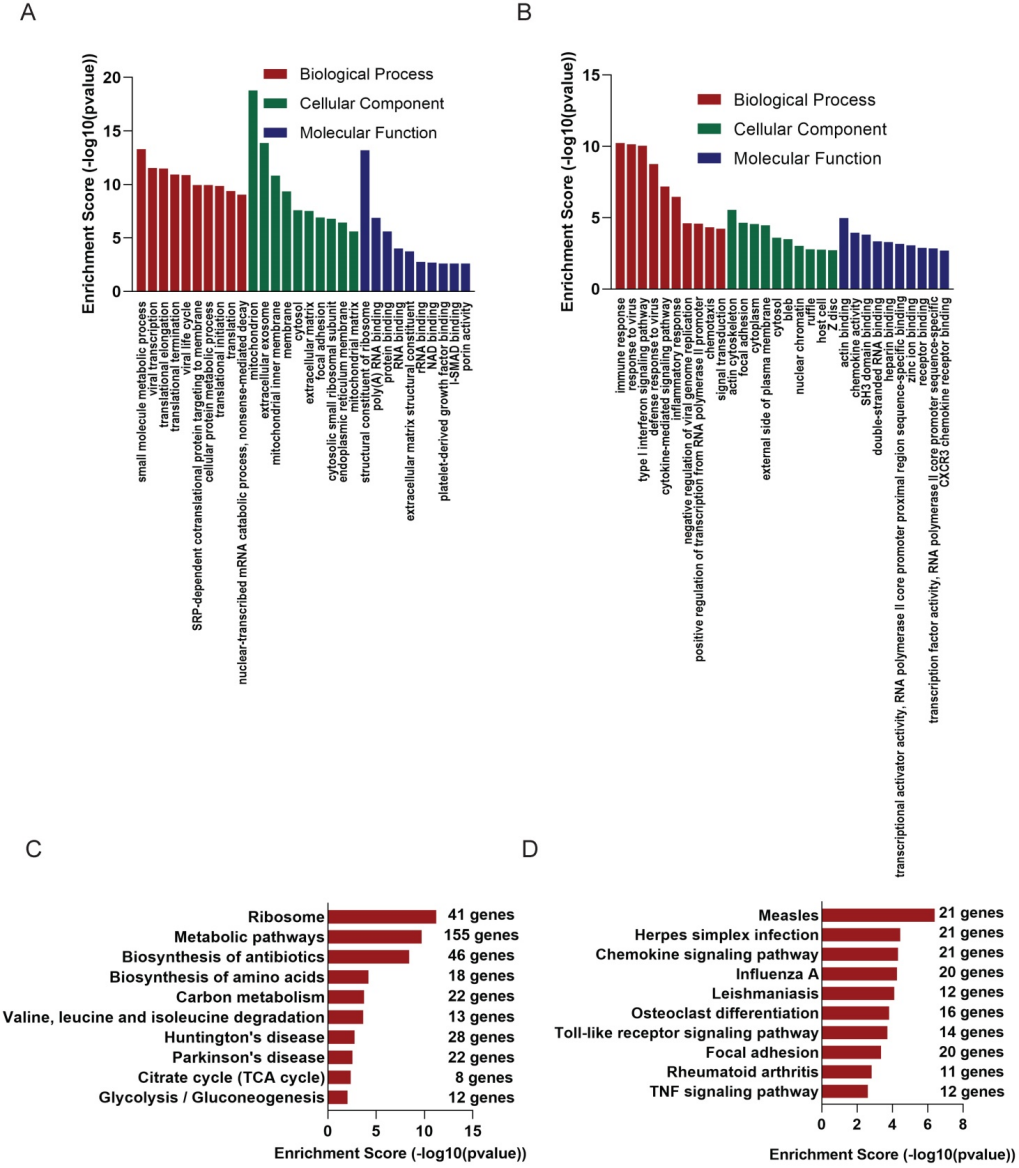
** GO and KEGG analysis of altered mRNAs.** The GO analysis of down-regulated mRNAs (A) and up-regulated mRNAs (B). KEGG pathways showed TOP 10 significantly enriched pathways of down-regulated mRNAs(C) and up-regulated mRNAs(D).

GO analysis of the altered 2221 mRNAs showed that up-regulated genes were enriched in immune response (ontology: biological process, GO:0006955), actin cytoskeleton (ontology: cellular component, GO:0015629) and actin binding (ontology: molecular function, GO:0003779) (Figure 3B); On the other hand, the top 10 enriched GO terms on down-regulated genes included small molecule metabolic process (ontology: biological process, GO:0044281), mitochondrion (ontology: cellular component, GO:0005739 and structural constituent of ribosome (ontology: molecular function, GO:0003735) (Figure 3A).

KEGG analysis was of these differentially expressed mRNAs was also conducted. Up-regulated mRNAs were involved in Measles, Herpes simplex infection, Chemokine signaling pathway (Figure 3D), while down-regulated mRNAs were most enriched in Ribosome, Metabolic pathways, Biosynthesis of antibiotics (Figure 3C).

### CNC network with GO and KEGG analyses

**Figure 4:**
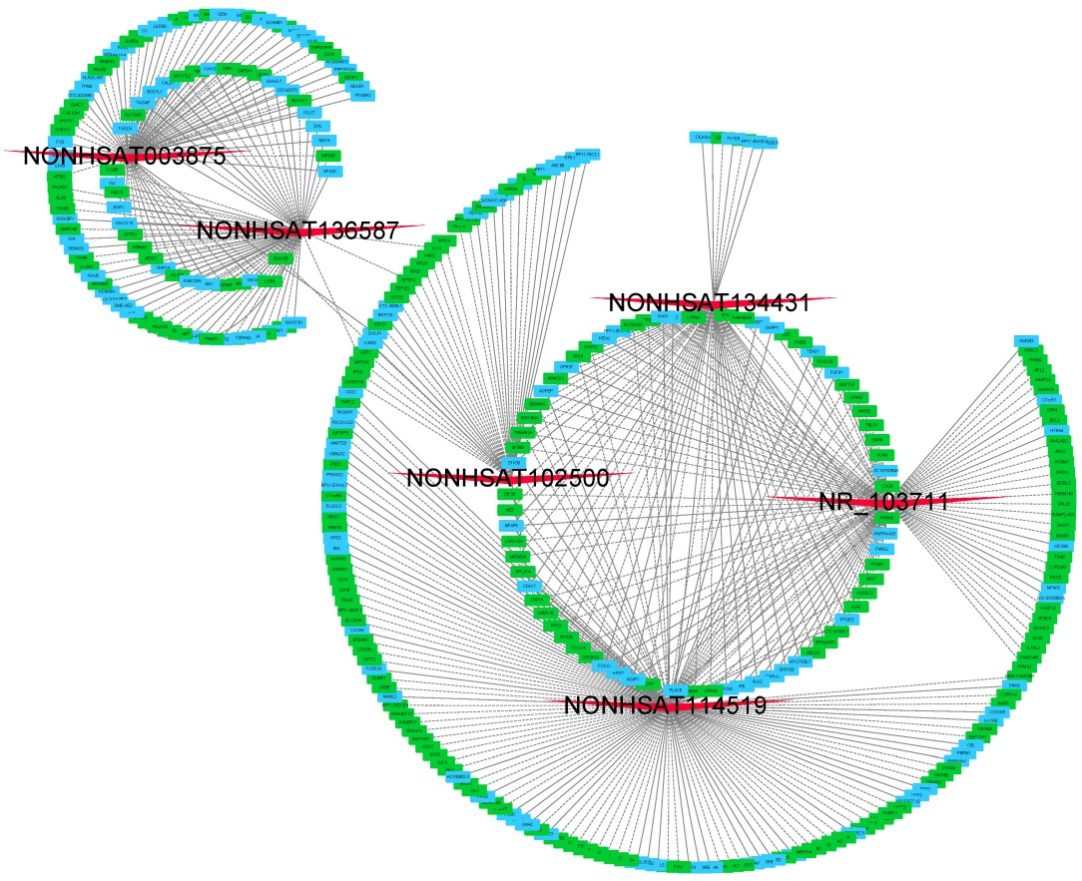
** CNC networks by validated lncRNAs.** The CNC networks were performed by 6 validated lncRNAs and 354 interacted mRNAs. The CNC networks were composed of 497 edges and 360 nodes with 207 positive interactions (continuous lines) and 290 negative interactions (dotted lines). The green nodes represented down-regulated mRNAs and blue ones denoted up-regulated mRNAs.

We then constructed lncRNA-mRNA co-expression networks on the basis of 6 validated lncRNAs as well as 354 mRNAs. In brief, the CNC networks were composed of 497 edges and 360 nodes with 207 positive interactions (continuous lines) and 290 negative interactions (dotted lines) (Figure 4). The lncRNA NONHSAT114519 is connected with 151 mRNAs, NR_103711 is connected with 97 mRNAs, NONHSAT003875 is connected with 77 mRNAs, NONHSAT136587, NONHSAT134431, and NONHSAT102500 are connected with 71, 66 and 35 mRNAs respectively.

**Figure 5:**
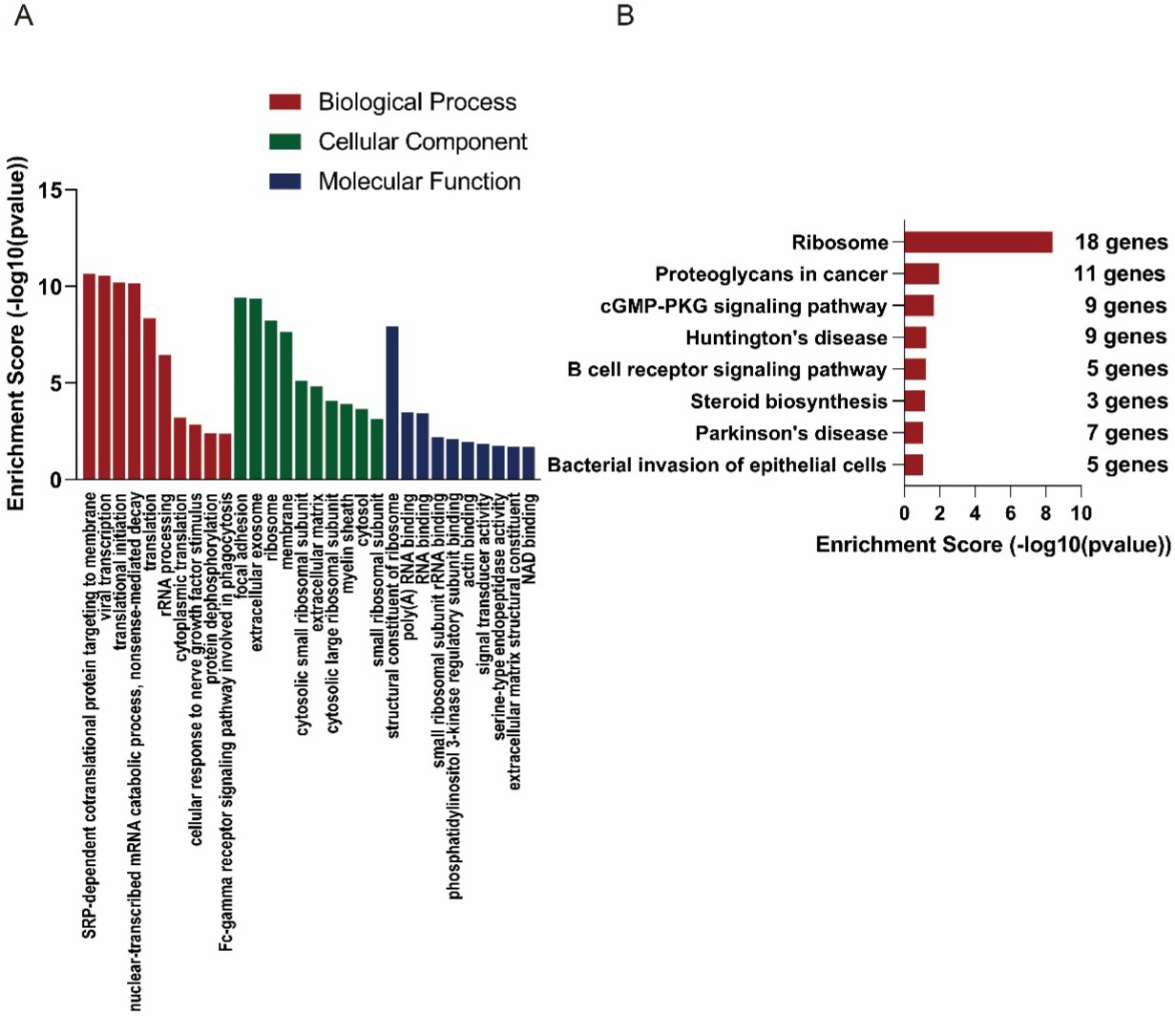
** GO and KEGG analyses of interacted mRNAs in CNC networks.** GO (A) and KEGG (B) analyses of CNC interacted mRNAs

In order to explore the functions of the lncRNAs, GO and KEGG analysis were also performed based on the interacted mRNAs from CNC networks. SRP-dependent cotranslational protein targeting to membrane (ontology: biological process, GO:0006614), focal adhesion (ontology: cellular component, GO:000592) and structural constituent of ribosome (ontology: molecular function, GO:0003735) were the most significantly enriched GO terms of the interacted genes (Figure 5A). KEGG data indicated that those targeted mRNAs were enriched in Ribosome, Proteoglycans in cancer, cGMP-PKG signaling pathway (Figure 5B).

## Discussion

A number of studies have demonstrated that lncRNAs play important roles in the differentiation 24, 25 and biological behaviors 26-32 of trophoblast cells. The impaired trophoblast migration and invasion abilities may further lead to preeclampsia and miscarriage. PSTT is originated from abnormally differentiated intermediate trophoblasts 2. However, aberrant lncRNAs expression profiles remain unknown in GTN, especially in PSTT. Presumably, the aberrantly expressed lncRNAs may contribute to the development and progression of PSTT. In the present study, expression levels of lncRNAs and mRNAs in four human PSTT tissues and four normal villi tissues were investigated by integrated microarray analysis. 2221 significantly altered mRNAs were identified (including 828 up-regulated; 1393 down-regulated) and 2260 differentially expressed lncRNAs (1247 upregulated; 1013 downregulated) in the PSTT compared to normal villi.

Our GO analysis showed that the most up-regulated mRNAs was involved in immune system process, while the most down-regulated mRNAs participate in mitochondrion.

Furthermore, the KEGG pathway analysis demonstrated that the aberrantly expressed mRNAs are mainly enriched in ribosome, metabolic pathways and biosynthesis of antibiotics (down-regulated mRNAs); measles, herpes simplex infection and chemokine signaling pathway (up-regulated mRNAs). Similarly, the most enriched GO terms are SRP-dependent cotranslational protein targeting to membrane, focal adhesion and structural constituent of ribosome of the lncRNA-interacted mRNAs. Furthermore, the top one enriched KEGG pathway is also ribosome. Such analysis inferred that PSTT might be mainly immune-regulated by ribosome. Besides, we also found that *HMOX1*, *EPHB2*, *CLIC4* and *CCL2* were up-regulated in PSTT, which were identified to be angiogenesis-related genes in previous studies 33-36. Protein-protein interaction network based on ten validated mRNAs were shown in Figure S3.

The gene expressions of our research were correlated with previous reported biomarkers including GATA3, which was known expressed in 71% of PSTT 37. Our results showed that compared to normal villi *MAPK14*, *MAPK13*, *MAPK7* and *MAPK1* were significantly down-regulated in PSTT, but Köbel et al. have reported that MAPK was highly expressed 38. PLAC8, also named onzin, is a small protein (∼16 kDa) that was originally described to be highly expressed in mouse placenta 39 and its deficiency in mice may result in innate immunity deficiency 40. We also found that PLAC8 is highly expressed in PSTT but not choriocarcinoma (Figure S2). Our previous study has indicated that PLAC8 may promote autophagic activity and improves the growth priority of human trophoblast cells 41. We have also demonstrated that the overexpression of AQPEP//LVRN could influence the invasion of trophoblast 42. In addition, NONHSAT003875/miR-363/EGR1 regulatory network in the carcinoma -associated fibroblasts was confirmed to control the angiogenesis of PSTT 43. Further studies focusing on these proteins may be promising methods to a better understanding of the pathogenesis process in PSTT.

To further study the changed signaling pathways between different types of GTN. GSEA analysis were performed on the basis of data set GSE135727. Significant differences in signaling pathways were identified by nominal p < 0.05 and FDR q-value < 0.25 (Table S1). Four representative GSEA-enrichment plots were indicated in Figure S4. Compared to PSTT, choriocarcinoma tended to alter in Coagulation, KRAS, P53 and MYC targets pathways. KRAS and P53 signaling pathways were significantly enriched in ETT and CC. GSEA analysis also suggest that alteration in the MYC targets pathway might be one of the major pathways altered in PSTT when compared to normal villi.

In conclusion, we revealed that lncRNAs and mRNAs are differentially expressed in PSTT and normal villi by microarray analysis. GO and KEGG showed that immunotherapy may be effective in PSTT. Furthermore, GO and KEGG analyses of lncRNA-interacted mRNAs based on 6 validated lncRNAs were also performed, which indicated that these specific lncRNAs may be involved in the biological processes of ribosome that might contribute to PSTT pathogenesis. GSEA analysis based on GSE135727 and microarray data set among different types GTN were performed to study the altered signaling pathways. The function of these lncRNAs in PSTT required further research.

## Supplementary Material

Supplementary figures and table.

## Figures and Tables

**Table 1:** The sequence of the primers for lncRNAs and mRNAs

Gene name	Forward and reverse primer	Tm (℃)	Product length (bp)
*DPP4*	F: 5'CTAGGGCAGGGACAGGATAA3'	60	126
	R: 5'TGTGAACAGCTCTTCTCCG3'		
*PARP14*	F: 5'ACATTGTGTGCCAGGTAG3'	60	116
	R: 5'GCTTCTTGCACTCTGAGC3'		
*ADAMTS9*	F: 5'AATGCTTTGAGTCTTTCCGA3'	60	112
	R: 5'GCTTCCCTTCATCAGCTTG3'		
	F: 5'ATGAATGCTGTCTGTGTGGAA3'	60	106
	R:5'CAGCAAAGAGTTGCCATATAGT3'		
*AQPEP*//*LVRN*	F: 5'CCATCAGCACATCTCCATTC3'	60	122
	R: 5'TTTACTCACAGCTTGCCAG3'		
*EGR1*	F: 5'CAAACCAATGGTGATCCTCTAT3'	60	101
	R: 5'CTGACACATGCTCTGAGAAT3'		
*PLK2*	F: 5'ATTAGTCAAGTGACGGTGC3'	60	115
	R: 5'GAAGGAGGTAGAGCCGAG3'		
*ADAMTS6*	F: 5'GATCTAATGCAGATGACTAGGC3'	60	107
	R: 5'ATTCCATGCTGATTGTCCAC3'		
*HTRA4*	F: 5'CCAATGCCCATGTTGTCAG3'	60	110
	R: 5'CACCGCAAGATCCAATTTAAG3'		
*CDH11*	F: 5'CCTGGGTCATTGTGACATA3'	60	101
	R: 5'CCTCTTCTGCTCAGAGACT3'		
*GAPDH*	F: 5'TGTTGCCATCAATGACCCCTT3'	60	201
	R: 5'CTCCACGACGTACTCAGCG3'		
NONHSAT003875	F: 5'GGTGGGGCAGAGAACATAGAAAAAGA3'	60	246
	R: 5'TCAAGGAAGAGTTGGGAAGGAAGAGA3'		
NONHSAT102500	F: 5'GGGAGCCTTTTCGTTTTGTGCTTTTT3'	58	202
	R: 5'ATTTCGTGCCCTTTGCCTCACTTTTC3'		
NONHSAT114519	F: 5'AAAGAGGTAGGAGCAAGAAAGAGGAG3'	58	214
	R: 5'TCTATGTGCATATTTGGGATGAGATT3'		
NONHSAT134431	F: 5'CATTATATAGATGGAAACATCGAGGG3'	60	264
	R: 5'TGCTTATTAGAATTTTTGTGGTGAGA3'		
NR_103711	F: 5'ACTGGACTGTGCAGTGTGGTTCTGAG3'	58	190
	R: 5'TGGGCATTTTGTTTATTTGTTTGGTG3'		
NONHSAT136587	F: 5'AAGAGATTTTGTCTAAAGGGCAGCAT3'	60	326
	R: 5'CAGAGAAAGCCAGTCGGTAAGTTCTG3'		

Tm: temperature. bp: base pair

**Table 2:** Top 20 significantly up-regulated and down-regulated lncRNAs

Probe Set ID	p	FC (abs)	Regulation	PSTT1	PSTT2	PSTT3	PSTT4	Villi1	Villi2	Villi3	Villi4	GeneSymbol	NonCodeID
TC0600002926.oe.1	2.876E-05	801.27435	up	14.57266	17.61935	14.21191	15.35068	5.161994	5.335738	5.820837	6.851423	lnc-LAMA4-4	NONHSAT114519
TC0500001030.oe.1	2.075E-05	734.45276	up	14.62951	16.50723	16.46539	13.9997	5.054627	5.078194	6.540246	6.846658	lnc-COMMD10-8	NONHSAT103347
TC0300003446.oe.1	0.0001658	650.9045	up	18.86095	18.16658	18.05667	18.81532	7.498871	7.042424	10.36552	11.6075	lnc-TNK2-9	NONHSAT094251
TC0300003445.oe.1	8.53E-05	460.6303	up	16.55804	15.36818	15.39982	16.55225	5.795643	5.417085	8.381965	8.893738	lnc-TNK2-10	NONHSAT094250
TC0500001029.oe.1	6.498E-05	330.9917	up	12.91026	15.37042	15.56427	12.90601	6.14006	4.675454	6.697259	5.755586	lnc-COMMD10-9	NONHSAT103345
TC0400000688.oe.1	5.345E-05	202.31433	up	14.13912	16.11971	14.57873	14.29385	6.061105	6.281838	7.472301	8.674349	lnc-COPS4-1	NONHSAT097204
TC0500002484.oe.1	0.0034374	105.16381	up	10.39773	11.63986	14.5663	7.646206	4.406109	4.058787	4.508239	4.410984	RP11-78C3.1	NONHSAT102500
TC0900000988.oe.1	0.0005695	100.53162	up	14.10921	16.4567	17.14165	16.3712	8.728636	7.745153	11.29517	9.703777	lnc-TRIM32-11	NONHSAT134431
TC0900002221.oe.1	0.0005368	99.72805	up	12.96074	14.52683	14.38401	14.13185	6.12572	5.533823	9.488853	8.295328	PAPPA-AS1	NR_103711
TC0400002102.oe.1	0.0004868	89.59299	up	11.60127	15.63266	14.06496	13.14861	6.616827	6.794709	6.660107	8.434599	lnc-LIN54-3	NONHSAT097205
TC0500002714.oe.1	0.001948	86.06065	up	10.93789	13.99877	12.5951	8.366644	4.825502	4.857947	5.668418	4.837413	lnc-ATG12-2	NONHSAT103341
TC1300001424.oe.1	0.0055827	71.04717	up	11.47673	10.99826	10.81088	16.38293	5.018981	5.45683	7.269685	7.320484	RP11-318G21.4	NONHSAT034434
TC0300003444.oe.1	9.85E-05	56.742313	up	12.1657	10.80384	11.8323	12.61162	5.355786	5.137434	6.225355	7.389472	lnc-TNK2-11	---
TC1400000340.oe.1	0.002576	54.023994	up	7.73035	11.65167	12.14659	8.160899	3.831198	4.072921	4.663983	4.099294	lnc-LRR1-6	NONHSAT036699
TC0400000156.oe.1	0.000826	53.37624	up	9.918715	12.84252	12.94642	13.54016	5.980694	5.678541	7.029627	7.606449	lnc-CPZ-1	---
TC1500001204.oe.1	0.000714	52.510838	up	12.26639	8.419676	12.00192	11.29106	4.842388	5.158345	5.542342	5.577796	RP11-73C9.1	NONHSAT040839
TC0100001151.oe.1	0.0125757	51.62064	up	12.92449	13.55079	13.03012	11.93119	6.584359	11.71653	4.420371	5.955827	lnc-IFI44-5	NONHSAT004079
TSUnmapped00002029.oe.1	0.0041549	51.021782	up	14.78756	9.150758	13.04631	11.74117	6.185886	7.775435	6.255181	5.817128	lnc-RTDR1-1	---
TC1200003068.oe.1	0.0001133	48.14622	up	9.09206	12.02009	10.97622	11.14791	5.561361	4.952483	5.211253	5.153783	lnc-HCAR3-1	NONHSAT031531
TC1600001762.oe.1	0.006927	43.289265	up	14.51361	12.54379	14.508	11.07535	8.799093	10.1918	6.002627	5.903477	lnc-KIAA0430-2	NONHSAT140763
TC0500002493.oe.1	0.0370893	288.59473	down	5.638968	5.017736	4.947758	5.209421	7.599888	8.595558	18.266	19.04404	lnc-EDIL3-6	NONHSAT102523
TC1100001750.oe.1	0.0011976	95.452	down	5.716751	6.666399	6.126871	5.764963	10.45444	10.97112	14.49622	14.66002	NONHSAG007410	NONHSAT017479
TC0200004232.oe.1	4.448E-05	75.3497	down	8.218021	9.405064	9.379367	9.757372	16.38943	16.15815	14.58752	14.56685	lnc-GCG-3	NONHSAT075191
TC0500002278.oe.1	5.254E-05	73.73616	down	7.36856	5.65632	6.768617	6.701671	11.46042	12.73857	13.50097	13.61241	lnc-CENPK-2	NONHSAT101743
TC0200004231.oe.1	6.523E-05	66.59564	down	8.589523	9.65343	9.179054	9.526411	16.27332	16.25944	14.06544	14.57964	lnc-GCG-4	NONHSAT075190
TC0Y00000312.oe.1	3.009E-05	48.222668	down	7.416726	7.051343	7.681014	7.152988	13.70421	13.75827	11.88662	12.31953	lnc-HSFY2-10	NONHSAT139611
TC0500000786.oe.1	0.0003598	45.157536	down	8.937391	8.656306	9.234922	11.02877	16.14978	15.59811	13.88346	14.21362	lnc-XRCC4-6	NONHSAT102522
TC1200000179.oe.1	0.013569	35.71296	down	8.367052	8.457984	10.00808	9.479094	17.09085	16.33409	11.90496	11.61581	lnc-RIMKLB-1	NONHSAT026363
TC0900001993.oe.1	0.0428375	34.091114	down	15.47735	10.6726	13.60676	6.988581	16.36274	14.92022	18.08391	17.74372	lnc-OMD-1	NONHSAT133188
TC14000659.hg.4	0.0052727	30.134325	down	6.758659	6.755126	8.207018	6.675376	10.21877	10.03378	13.81844	13.97853	MEG8	NR_024149
TC1200000178.oe.1	0.0203783	25.076464	down	6.339385	7.046954	8.730094	8.730531	14.91692	14.49464	10.01952	10.00893	lnc-RIMKLB-3	NONHSAT026360
TC1400000032.oe.1	4.427E-05	23.937801	down	5.908465	6.809318	6.180269	6.861191	10.77885	10.08512	11.60237	11.61778	lnc-APEX1-3	NONHSAT035586
TC0800000155.oe.1	0.0219285	21.791851	down	7.052204	6.485874	7.214129	7.360553	13.46182	14.35795	8.499699	9.576158	lnc-PDGFRL-3	NONHSAT125277
TSUnmapped00000268.oe.1	0.0018863	21.46142	down	6.592407	6.48301	7.71503	7.08154	10.66954	9.543282	12.30839	13.04547	lnc-DLK1-5	---
TC1400000966.oe.1	0.0049216	20.716341	down	6.727886	6.515725	6.867692	6.953129	9.094263	9.745587	12.64482	13.07055	OTTHUMG00000029060	NONHSAT039835
TC0500001872.oe.1	0.0040102	19.861301	down	9.433871	6.534947	7.719427	6.949482	13.23098	13.10029	10.4047	11.14931	lnc-TAS2R1-24	NONHSAT100322
TC0300002485.oe.1	0.0014715	19.597805	down	7.21331	7.91722	7.539716	7.546046	13.63197	12.58882	10.73676	10.42922	lnc-PRICKLE2-6	NONHSAT090260
TC0800000049.oe.1	1.843E-05	18.799055	down	7.130774	5.713998	6.799021	6.786564	11.20656	10.67594	10.49293	10.98528	lnc-MCPH1-3	NONHSAT124787
TC0600001704.oe.1	0.0002245	17.906603	down	6.236846	4.643425	6.369543	5.58888	9.445736	9.72422	10.91174	9.406675	lnc-SLC22A1-6	NONHSAT115901
TSUnmapped00000213.oe.1	0.006309	17.698973	down	6.089841	7.533654	7.496603	6.496414	13.19711	12.04852	9.260924	9.692332	lnc-COLEC10-1	---

P values were calculated using unpaired t-test and adjusted for multiple comparison. FC (abs): the absolute ratio (no log scale) of average normalized intensities between PSST and normal villi. PSTT 1 to 4 and Villi 1 to 4: each sample's normalized intensity (log2 scale). The same goes for the follow-up table.

**Table 3:** Top 20 significantly up-regulated and down-regulated mRNAs

Probe Set ID	p	FC (abs)	Regulation	PSTT1	PSTT2	PSTT3	PSTT4	Villi1	Villi2	Villi3	Villi4	GeneSymbol	strand
TC05000549.hg.4	3.19235E-05	591.0085	up	14.27506	16.42701	15.97555	13.53596	4.718653	5.511204	6.863236	6.292351	*AQPEP*	+
TC08000297.hg.4	0.004722227	458.7278	up	17.73262	18.95313	17.89105	17.81702	5.701577	6.169894	11.17874	13.97764	*HTRA4*	+
TC04001304.hg.4	0.001240178	298.35522	up	17.31657	11.57826	13.04546	16.06837	5.285185	7.790433	5.599919	6.449578	*CXCL9*	-
TC05001550.hg.4	0.001369429	220.83633	up	10.24931	11.60825	14.99216	8.492987	3.193323	3.266655	3.947432	3.787961	*RP11-78C3.1*	-
TC02004950.hg.4	0.000845117	215.45534	up	16.68137	12.53434	15.61682	14.56816	7.122076	9.626941	5.731162	5.915533	*IGKV3-15*	-
TC04001753.hg.4	0.000546863	160.13506	up	13.51392	17.16884	15.11037	15.33165	7.005078	6.216195	9.034913	9.57601	*HPGD*	-
TC21000287.hg.4	0.000300168	154.46967	up	10.59372	13.89927	14.52517	11.97499	4.889561	4.769378	6.180188	6.069306	*AF127936.7*	-
TC06000935.hg.4	0.002012451	119.142746	up	15.03825	12.59383	14.74779	11.78778	7.760329	8.965266	5.600394	4.255473	*PLN*	+
TC04001344.hg.4	0.000890978	111.79973	up	12.05535	15.73258	14.5653	13.71613	6.178575	5.559375	8.119697	8.99262	*PLAC8*	-
TC01002774.hg.4	0.001239457	107.338455	up	14.57023	12.06937	14.90409	11.53205	7.059074	8.57903	5.266312	5.187234	*PTGER3*	-
TC09001525.hg.4	0.000788934	105.381905	up	12.49726	13.83948	13.67676	13.49805	5.165512	4.803937	9.22176	7.44241	*PAPPA-AS1*	-
TC04001270.hg.4	0.004180308	93.43535	up	14.72628	7.911443	11.8912	10.37814	4.447676	5.532782	4.042093	4.700925	*IGJ*	-
TC06001773.hg.4	0.000682254	90.94472	up	17.52071	17.21107	17.07343	17.87255	12.42595	11.83653	11.40955	7.978059	*PLA2G7*	-
TC09000038.hg.4	1.45655E-05	89.93031	up	12.54376	13.83348	11.77462	13.4532	6.069004	6.997876	6.103081	6.472157	*PDCD1LG2*	+
TC08000150.hg.4	0.001507328	77.45381	up	9.458164	13.66541	9.141149	12.47632	4.575369	4.467356	5.268524	5.328737	*AC100802.3*	+
TC16000898.hg.4	0.004886279	72.98015	up	15.8717	13.48058	15.87599	12.17157	9.269753	10.68977	6.477879	6.204714	*MYH11*	-
TC02004978.hg.4	0.007539266	68.028145	up	12.40944	5.704045	9.07485	11.44005	3.022104	4.692099	3.458396	3.103549	*IGKV2D-28*	+
TC09000593.hg.4	0.003252797	66.812675	up	13.59354	16.24804	15.87841	15.89478	7.747241	7.059897	11.45904	11.10039	*PAPPA*	+
TC04001305.hg.4	0.003304237	56.946953	up	13.42061	10.216	9.274749	13.00267	5.717486	7.589098	4.4457	4.835558	*CXCL10*	-
TC07001008.hg.4	0.006718533	56.503063	up	8.288177	11.47205	10.50508	14.01944	5.617546	7.370414	3.747692	4.268066	*AOC1*	+
TC05001804.hg.4	1.13295E-05	1304.5027	down	4.789694	4.781364	4.235359	4.61926	16.16161	16.39945	13.47281	13.78895	*CXCL14*	-
TC09000799.hg.4	0.018049004	212.94751	down	4.27957	5.484641	4.4404	5.481874	16.75124	16.76882	8.093913	9.009928	*PAEP*	+
TC02002478.hg.4	6.23932E-05	97.702	down	9.555952	11.27693	10.58138	11.23763	18.28535	18.10812	16.43987	16.25981	*DPP4*	-
TC07000722.hg.4	8.31021E-05	84.22738	down	5.371543	4.937615	5.333688	5.770316	13.12773	12.60029	10.39067	10.87934	*MET*	+
TC05001406.hg.4	1.12984E-05	81.3294	down	6.72622	5.453224	6.382895	5.675251	11.48571	12.12078	12.89001	13.12391	*ADAMTS6*	-
TC04001335.hg.4	2.90216E-05	67.747826	down	6.126198	4.339462	4.120981	5.204585	11.40271	11.55877	10.85734	10.30082	*TMEM150C*	-
TC12000624.hg.4	0.004995465	61.509308	down	4.402699	5.301405	3.562141	4.060987	7.529198	8.502194	12.1875	12.87927	*LGR5*	+
TC17001112.hg.4	4.01987E-06	50.456596	down	5.316912	5.662082	5.71819	5.521931	11.64679	11.93939	10.48815	10.77267	*SLC25A35*	-
TC11001274.hg.4	0.002174686	49.247604	down	6.986232	9.491581	7.071369	8.253414	11.63622	12.38722	14.7717	15.49538	*INS*	-
TC14000407.hg.4	0.001220558	48.467384	down	5.233055	4.43302	5.984023	6.457338	10.24269	9.109385	12.45468	12.69645	*PLEKHH1*	+
TC11001505.hg.4	0.003624685	47.256374	down	5.270397	8.929316	6.762051	5.760069	12.42111	10.82543	11.03148	14.69356	*MUC15*	-
TC17001474.hg.4	0.01790399	47.178997	down	6.29473	6.818648	8.245532	6.971005	11.13493	8.638376	15.39309	15.40381	*KRT23*	-
TC09001334.hg.4	0.042527948	46.31269	down	15.78603	11.14224	14.07409	6.869641	16.99306	15.18752	19.40821	18.41655	*OGN*	-
TC05001151.hg.4	0.000906728	45.62948	down	9.584321	6.191512	8.45069	8.70398	14.70294	14.62603	12.47793	13.17118	*SEMA5A*	-
TC13000335.hg.4	0.035226125	45.062977	down	11.85807	7.952482	11.39816	3.792094	15.23736	15.66917	12.14623	13.92353	*CLDN10*	+
TC16000223.hg.4	0.007389613	43.19478	down	4.741453	4.390289	4.657523	4.420307	12.78836	11.86416	7.820678	7.467516	*ACSM3*	+
TC08000022.hg.4	7.42939E-06	42.766193	down	8.376294	7.954287	9.139468	9.321456	14.22422	13.52475	14.43939	14.27674	*AGPAT5*	+
TC02000293.hg.4	0.040336728	39.249073	down	7.892764	5.327816	7.782251	6.065664	15.51643	15.24631	8.215117	9.268984	*EPCAM*	+
TC01002886.hg.4	0.000537544	37.86066	down	6.817955	5.422742	6.118052	6.653155	9.519774	11.35453	12.57758	12.53053	*F3*	-
TC14000056.hg.4	0.000102863	37.460266	down	6.568504	8.084141	6.370689	7.934472	12.03227	11.67531	12.92644	13.23294	*PNP*	+

## Abbreviations

PSTT: placental site trophoblastic tumor
RT-qPCR: reverse transcription and quantitative real-time polymerase reaction
GO: Gene Ontology
KEGG: Kyoto Encyclopedia of Genes and Genomes
CNC: coding-non-coding gene co-expression
PCCs: Pearson correlation coefficients
SRP: signal-recognition particle
GTN: gestational trophoblastic neoplasms
lncRNAs: long non-coding RNAs
GSEA: gene set enrichment analysis
ETT: epithelioid trophoblastic tumor
CC: choriocarcinoma
PCA: principal component analysis

## References

[1] Kurman RJ, Scully RE, Norris HJ (1976). Trophoblastic pseudotumor of the uterus: an exaggerated form of "syncytial endometritis" simulating a malignant tumor. Cancer.

[2] Feng X, Wei Z, Zhang S, Du Y, Zhao H (2019). A Review on the Pathogenesis and Clinical Management of Placental Site Trophoblastic. Frontiers in oncology.

[3] Feng X, Wei Z, Zhang S, Du Y, Zhao H (2019). A Review on the Pathogenesis and Clinical Management of Placental Site Trophoblastic. Frontiers in oncology.

[4] Seckl MJ, Sebire NJ, Fisher RA, Golfier F, Massuger L, Sessa C (2013). Gestational trophoblastic disease: ESMO Clinical Practice Guidelines for diagnosis, treatment and follow-up. Annals of oncology: official journal of the European Society for Medical Oncology.

[5] Schmid P, Nagai Y, Agarwal R, Hancock B, Savage PM, Sebire NJ, Lindsay I, Wells M, Fisher RA, Short D, Newlands ES, Wischnewsky MB, Seckl MJ (2009). Prognostic markers and long-term outcome of placental-site trophoblastic tumours: a retrospective observational study. Lancet (London, England).

[6] Lan C, Li Y, He J, Liu J (2010). Placental site trophoblastic tumor: lymphatic spread and possible target markers. In.

[7] Papadopoulos AJ, Foskett M, Seckl MJ, McNeish I, Paradinas FJ, Rees H, Newlands ES (2002). Twenty-five years' clinical experience with placental site trophoblastic tumors. The Journal of reproductive medicine.

[8] Hassadia A, Gillespie A, Tidy J, Everard R G N J, Wells M, Coleman R, Hancock B (2005). Placental site trophoblastic tumour: clinical features and management. Gynecologic oncology.

[9] Zhao J, Lv WG, Feng FZ, Wan XR, Liu JH, Yi XF, Qu PP, Xue FX, Wu YM, Zhao X, Ren T, Yang JJ, Xie X, Xiang Y (2016). Placental site trophoblastic tumor: A review of 108 cases and their implications for prognosis and treatment. Gynecologic oncology.

[10] Lee H, Shin W, Jang YJ, Choi CH, Lee J, Bae D, Kim B (2018). Clinical characteristics and outcomes of placental site trophoblastic tumor: experience of single institution in Korea. Obstetrics & gynecology science.

[11] Braga A, Mora P, de Melo AC, Nogueira-Rodrigues A, Amim-Junior J, Rezende-Filho J, Seckl MJ (2019). Challenges in the diagnosis and treatment of gestational trophoblastic neoplasia worldwide. In.

[12] Wang C, Zhu C, Xu J, Wang M, Zhao W, Liu Q, Zhao G, Zhang Z (2019). The lncRNA UCA1 promotes proliferation, migration, immune escape and inhibits apoptosis in gastric cancer by sponging anti-tumor miRNAs. Molecular Cancer.

[13] Yang L, Lin C, Jin C, Yang JC, Tanasa B, Li W, Merkurjev D, Ohgi KA, Meng D, Zhang J, Evans CP, Rosenfeld MG (2013). lncRNA-dependent mechanisms of androgen-receptor-regulated gene activation programs. Nature.

[14] Sun X, Yuan Y, Xiao Y, Lu Q, Yang L, Chen C, Guo Q (2018). Long non-coding RNA, Bmcob, regulates osteoblastic differentiation of bone marrow mesenchymal stem cells. Biochemical and biophysical research communications.

[15] Wambecke A, Ahmad M, Morice P, Lambert B, Weiswald L, Vernon M, Vigneron N, Abeilard E, Brotin E, Figeac M, Gauduchon P, Poulain L, Denoyelle C, Meryet-Figuiere M (2021). The lncRNA 'UCA1' modulates the response to chemotherapy of ovarian cancer through. Molecular oncology.

[16] Thakur KK, Kumar A, Banik K, Verma E, Khatoon E, Harsha C, Sethi G, Gupta SC, Kunnumakkara AB (2021). Long noncoding RNAs in triple-negative breast cancer: A new frontier in the. Journal of cellular physiology.

[17] Xu W, Ding M, Wang B, Cai Y, Guo C, Yuan C (2021). Molecular mechanism of the canonical oncogenic lncRNA MALAT1 in gastric cancer. Current medicinal chemistry.

[18] Wang X, Li X, Zhou Y, Huang X, Jiang X (2021). Long non-coding RNA OIP5-AS1 inhibition upregulates microRNA-129-5p to repress. Cell biology and toxicology.

[19] Zhang M, Gao F, Yu X, Zhang Q, Sun Z, He Y, Guo W (2021). LINC00261: a burgeoning long noncoding RNA related to cancer. Cancer Cell International.

[20] Wang ZK, Yang L, Wu LL, Mao H, Zhou YH, Zhang PF, Dai GH (2017). Long non-coding RNA LINC00261 sensitizes human colon cancer cells to cisplatin therapy. Brazilian journal of medical and biological research = Revista brasileira de pesquisas medicas e biologicas.

[21] Cho EJ, Chun S, Park H, Sung CO, Kim K (2020). Whole transcriptome analysis of gestational trophoblastic neoplasms reveals altered PI3K signaling pathway in epithelioid trophoblastic tumor. Gynecologic Oncology.

[22] Edgar R, Domrachev M, Lash AE (2002). Gene Expression Omnibus: NCBI gene expression and hybridization array data repository. Nucleic acids research.

[23] Chen C, Chen H, Zhang Y, Thomas HR, Frank MH, He Y, Xia R (2020). TBtools: An Integrative Toolkit Developed for Interactive Analyses of Big Biological Data. Molecular plant.

[24] Chu T, Mouillet J, Cao Z, Barak O, Ouyang Y, Sadovsky Y (2021). RNA Network Interactions During Differentiation of Human Trophoblasts. Frontiers in Cell and Developmental Biology.

[25] You J, Wang W, Chang H, Yi Y, Zhao H, Zhu H, Sun Y, Tang M, Wang C, Sang Y, Feng G, Cheng S, Leung PCK, Zhu Y (2021). The BMP2 Signaling Axis Promotes Invasive Differentiation of Human Trophoblasts. Frontiers in cell and developmental biology.

[26] Wang R, Zou L, Yang X (2021). microRNA-210/ Long non-coding RNA MEG3 axis inhibits trophoblast cell migration and invasion by suppressing EMT process. Placenta.

[27] Chen X, Guo D, Yin T, Yang J (2021). Non-Coding RNAs Regulate Placental Trophoblast Function and Participate in Recurrent Abortion. Frontiers in Pharmacology.

[28] Chi Z, Gao Q, Sun Y, Zhou F, Wang H, Shu X, Zhang M (2021). LINC00473 downregulation facilitates trophoblast cell migration and invasion via the. Environmental toxicology.

[29] Fan X, Lou J, Zheng X, Wang Y, Wang J, Luo M, Hu M (2021). Interference with lncRNA NEAT1 promotes the proliferation, migration, and invasion. Immunopharmacology and immunotoxicology.

[30] Xie J, Liang T, Zhao J, Xu Z, Tian P, Wang R, Mi C, Huang W, Chen W, Zhang H (2021). Lnc-HZ08 regulates BPDE-induced trophoblast cell dysfunctions by promoting PI3K. Cell biology and toxicology.

[31] Xu Z, Tian P, Guo J, Mi C, Liang T, Xie J, Huang W, Dai M, Chen W, Zhang H (2021). Lnc-HZ01 with m6A RNA methylation inhibits human trophoblast cell proliferation and. The Science of the total environment.

[32] Chen Y, Li Z, Chen X, Zhang S (2021). Long non-coding RNAs: From disease code to drug role. Acta pharmaceutica Sinica. B.

[33] Luu Hoang KN, Anstee JE, Arnold JN (2021). The Diverse Roles of Heme Oxygenase-1 in Tumor Progression. Frontiers in immunology.

[34] Sato S, Vasaikar S, Eskaros A, Kim Y, Lewis JS, Zhang B, Zijlstra A, Weaver AM (2019). EPHB2 carried on small extracellular vesicles induces tumor angiogenesis via. JCI insight.

[35] Edwards JC, Bruno J, Key P, Cheng Y (2014). Absence of chloride intracellular channel 4 (CLIC4) predisposes to acute kidney. BMC nephrology.

[36] Bonapace L, Coissieux M, Wyckoff J, Mertz KD, Varga Z, Junt T, Bentires-Alj M (2014). Cessation of CCL2 inhibition accelerates breast cancer metastasis by promoting angiogenesis. Nature.

[37] Banet N, Gown AM, Shih I, Kay Li Q, Roden RBS, Nucci MR, Cheng L, Przybycin CG, Nasseri-Nik N, Wu L, Netto GJ, Ronnett BM, Vang R (2015). GATA-3 expression in trophoblastic tissues: an immunohistochemical study of 445 cases, including diagnostic utility. In.

[38] Köbel M, Pohl G, Schmitt WD, Hauptmann S, Wang T, Shih I (2005). Activation of Mitogen-Activated Protein Kinase Is Required for Migration and Invasion of Placental Site Trophoblastic Tumor. The American Journal of Pathology.

[39] Tatura M, Schmidt H, Haijat M, Stark M, Rinke A, Diels R, Lawlor RT, Scarpa A, Schrader J, Hackert T, Schimmack S, Gress TM, Buchholz M (2020). Placenta-Specific 8 Is Overexpressed and Regulates Cell Proliferation in Low-Grade Human Pancreatic Neuroendocrine Tumors. Neuroendocrinology.

[40] Ledford JG, Kovarova M, Koller BH (2007). Impaired host defense in mice lacking ONZIN. Journal of immunology (Baltimore, Md.: 1950).

[41] Feng X, Wei Z, Tao X, Du Y, Wu J, Yu Y, Yu H, Zhao H (2021). PLAC8 promotes the autophagic activity and improves the growth priority of human trophoblast cells. The FASEB Journal.

[42] Feng X, Wei Z, Zhang S, Zhou J, Wu J, Luan B, Du Y, Zhao H (2021). Overexpression of LVRN impedes the invasion of trophoblasts by inhibiting epithelial-mesenchymal transition. Acta Biochimica et Biophysica Sinica.

[43] Zhang S, Tao X, Cao Q, Feng X, Wu J, Yu H, Yu Y, Xu C, Zhao H (2020). lnc003875/miR-363/EGR1 regulatory network in the carcinoma -associated fibroblasts controls the angiogenesis of human placental site trophoblastic tumor (PSTT). Experimental Cell Research.

