# Supplementary material for: Expression profiling of lncRNAs and mRNAs in placental site trophoblastic tumor (PSTT) by microarray
Source: Int J Med Sci. 2022 Jan 1;19(1):1–12. doi: 10.7150/ijms.65002 (PMC8692111; doi:10.7150/ijms.65002)
Supplement: Supplementary file 1 — Supplementary figures and table. [file ijmsv19p0001s1.pdf]

Supplemental materials

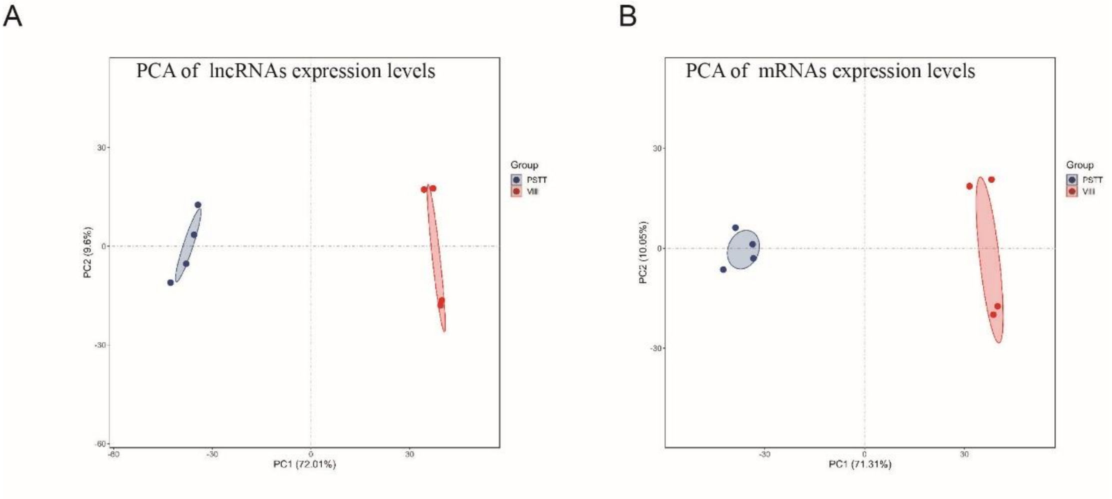

Figure S1 Principal component analysis (PCA) of the identified differentially expressed lncRNAs and mRNAs. PCA of eight distinct samples according to the expression levels of the mRNA (B) and lncRNA (A).

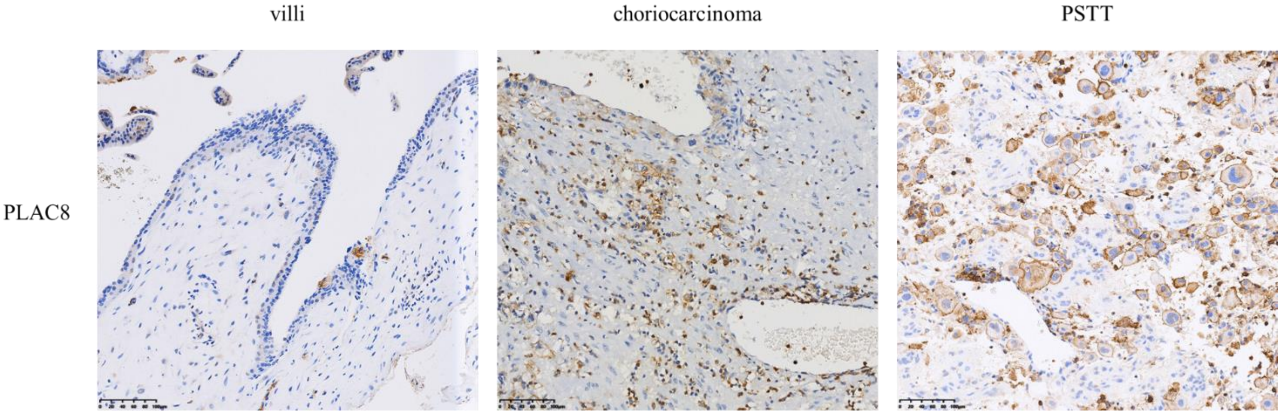

Figure S2 PLAC8 was expressed in normal villi, choriocarcinoma and PSTT. PLAC8 was slightly positive in normal villi, but partly positive in choriocarcinoma and diffusely expressed in PSTT. Scale bar = 100μm.

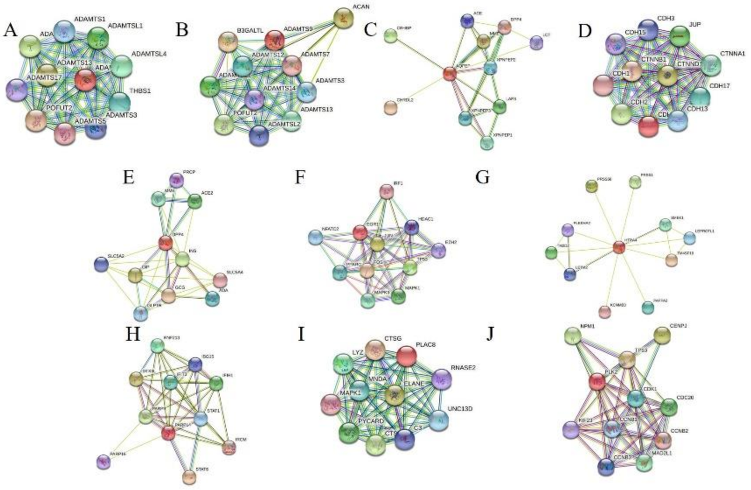

Figure S3 Protein-protein interaction networks based on ten validated mRNAs. Protein-protein networks of ten validated 10 mRNAs. A-J represents ADAMTS6, ADAMTS9, AQPEP, CDH1, DPP4, EGR1, HTRA4, PARP14, PLAC8 and PLK2 respectively.

Table S1 Enriched pathways of ETT vs PSTT, PSTT vs CC, CC vs ETT, CC vs PSTT and PSTT vs Villi.

		NAME	SIZE	NES	NOM p value	FDR q value
GSE135727	ETT vs PSTT	HALLMARK_HEDGEHOG_SIGNALING	35	1.62	0.000	0.038
		HALLMARK_APICAL_SURFACE	43	1.55	0.037	0.103
		HALLMARK_ESTROGEN_RESPONSE_LATE	195	1.4	0.000	0.214
		HALLMARK_KRAS_SIGNALING_DN	188	1.38	0.000	0.181
		HALLMARK_APICAL_JUNCTION	194	1.35	0.045	0.171
		HALLMARK_COAGULATION	136	1.34	0.000	0.162
		HALLMARK_PANCREAS_BETA_CELLS	40	1.34	0.048	0.150
		HALLMARK_P53_PATHWAY	190	1.34	0.036	0.140
		HALLMARK_GLYCOLYSIS	195	1.33	0.022	0.133
		HALLMARK_HEME_METABOLISM	190	1.32	0.000	0.120
		HALLMARK_PI3K_AKT_MTOR_SIGNALING	104	1.31	0.048	0.112
		HALLMARK_IL2_STAT5_SIGNALING	194	1.3	0.000	0.125
	PSTT vs CC	HALLMARK_UV_RESPONSE_DN	137	1.47	0.006	0.097
	CC vs ETT	HALLMARK_COAGULATION	136	1.66	0.000	0.053
	CC vs PSTT	HALLMARK_HYPOXIA	190	1.76	0.000	0.014
		HALLMARK_E2F_TARGETS	187	1.67	0.000	0.021
		HALLMARK_UV_RESPONSE_UP	152	1.64	0.000	0.019
		HALLMARK_MYC_TARGETS_V2	58	1.63	0.005	0.019
		HALLMARK_KRAS_SIGNALING_DN	188	1.59	0.000	0.025
		HALLMARK_G2M_CHECKPOINT	184	1.58	0.000	0.024
		HALLMARK_COAGULATION	136	1.58	0.001	0.021
		HALLMARK_WNT_BETA_CATENIN_SIGNALING	42	1.52	0.029	0.034
		HALLMARK_IL2_STAT5_SIGNALING	194	1.48	0.006	0.045
		HALLMARK_MTORC1_SIGNALING	192	1.48	0.003	0.043
		HALLMARK_MYC_TARGETS_V1	188	1.48	0.001	0.040
		HALLMARK_ESTROGEN_RESPONSE_LATE	195	1.46	0.007	0.043
		HALLMARK_XENOBIOTIC_METABOLISM	197	1.43	0.008	0.057
		HALLMARK_UNFOLDED_PROTEIN_RESPONSE	106	1.43	0.023	0.055
		HALLMARK_P53_PATHWAY	190	1.42	0.007	0.057
		HALLMARK_TNFA_SIGNALING_VIA_NFKB	197	1.4	0.013	0.061
		HALLMARK_ESTROGEN_RESPONSE_EARLY	192	1.36	0.027	0.090
		HALLMARK_GLYCOLYSIS	195	1.34	0.022	0.100
		HALLMARK_KRAS_SIGNALING_UP	193	1.29	0.045	0.145
microarray data	PSTT vs Villi	HALLMARK_MYC_TARGETS_V2	52	1.43	0.035	0.146

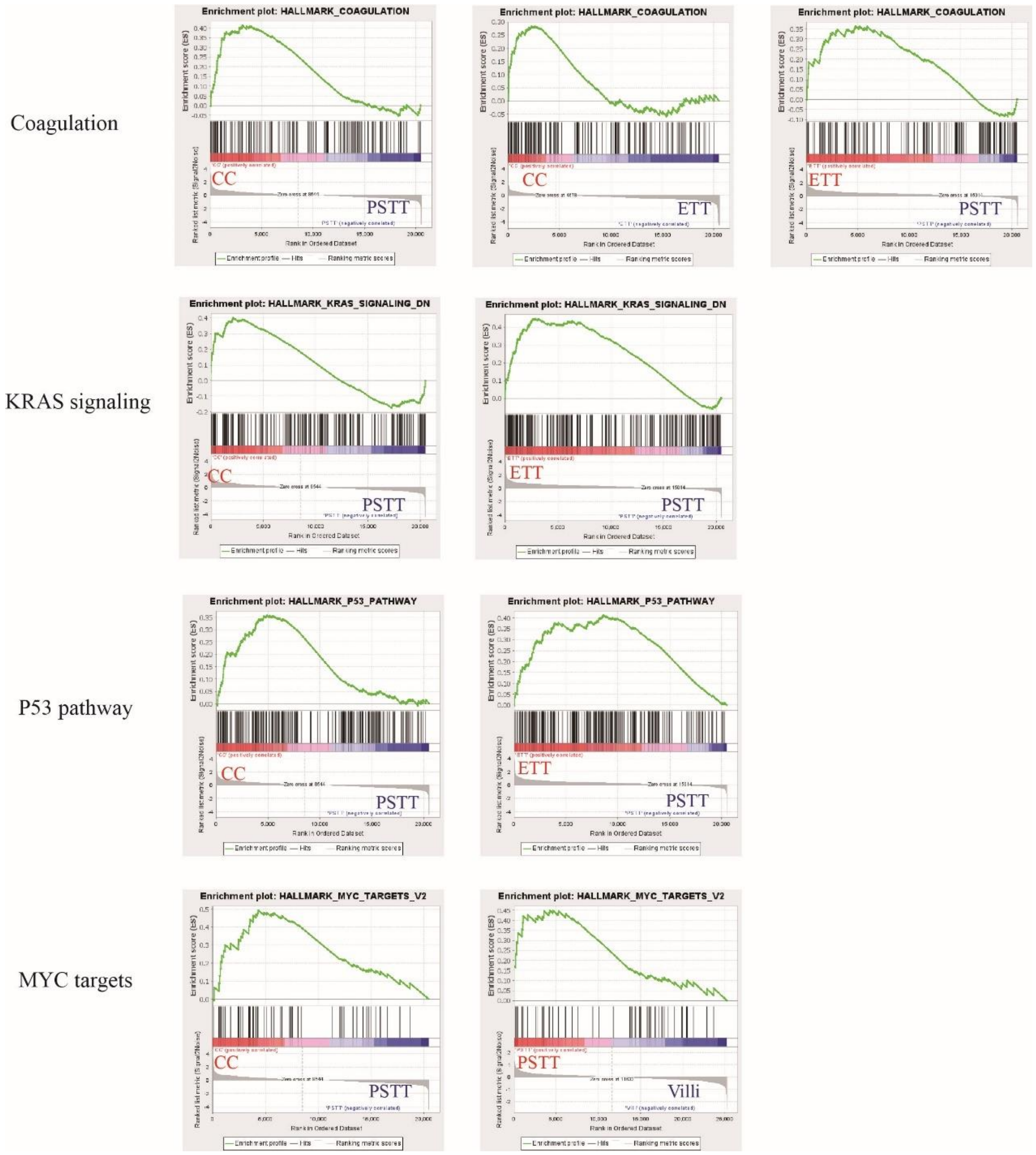

Figure S4 Enrichment score distribution of Coagulation pathway, KRAS signaling pathway, p53 pathway and MYC targets pathway among CC, PSTT, ETT and normal villi.
